# Hybrid repair with soft elephant trunk for acute type B dissection in a patient with right-sided aortic arch and Kommerell diverticulum

**DOI:** 10.1016/j.xjse.2025.100044

**Published:** 2025-01-27

**Authors:** Veronica Lorenz, Luigi Muzzi, Eugenio Neri

**Affiliations:** Cardiac Surgery-Aortic Unit, Università degli Studi di Siena, Siena, Italy

**Figure:**
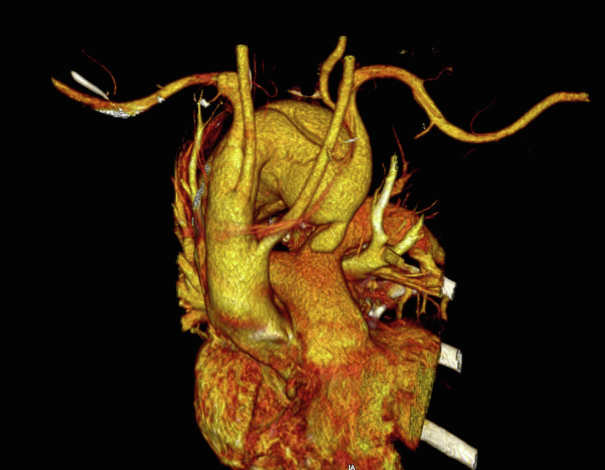
Right-sided aortic arch, aberrant left subclavian and Kommerell diverticulum.

**Central Message**

A complex hybrid approach can be used to treat type B dissection that occurs in the rare anatomical setting of right-sided aortic arch with aberrant left subclavian artery and Kommerell diverticulum.

Right-sided aortic arch (RSAA) is a rare congenital anomaly, occurring in less than 0.1% of the population,^1^ often accompanied by aberrant left subclavian artery (ALSA) originating from Kommerell diverticulum (KD).[Bibr bib6][Bibr bib7] These cases are infrequently documented in the literature, and no standardized treatment guidelines exist. Herein, we describe a successful 2-step hybrid surgical approach for an acute type B aortic dissection originating from a large KD in a patient with an RSAA and ALSA. The patient gave the written consent to publish the article; institutional review board approval not required.

## Case Presentation

A 54-year-old male patient presented to the emergency department with sudden-onset chest pain. Computed tomography imaging revealed an acute type B aortic dissection with a RSAA, ALSA, and a large KD. Given the patient's anatomical complexity and ongoing pain, urgent surgical intervention was necessary. A single endovascular procedure was deemed unsuitable because of the complexity of the case. Thus, a hybrid 2-step strategy was selected, combining a soft elephant trunk surgery and endovascular technique.

## Surgical Procedure

Under general anesthesia and with the patient in a supine position, a gooseneck catheter was inserted into the true lumen of the distal arch using angioscopic guidance (Video 1) . After sternotomy, the arch vessels were exposed and cardiopulmonary bypass was initiated with cannulation of the ascending aorta and right atrium. Under moderate hypothermic circulatory arrest with bilateral antegrade cerebral perfusion, the aorta was transected between the left carotid and right subclavian arteries. A 20- × 150-mm Dacron graft was inserted into the true lumen using the gooseneck catheter, with anastomosis to the aortic neck. A second 20-mm branched graft was connected to the aorta and the elephant trunk. After 22 minutes of circulatory arrest, antegrade systemic perfusion was restored through a graft perfusion branch. The supra-aortic vessels were then sequentially reimplanted using the limbs of the branched graft. Lastly, flow was restored to the left axillary artery using an extra-anatomical bypass tunneled through the second intercostal space to the left common carotid.

Two days postsurgery, a follow-up computed tomography scan showed the correct implant of the soft elephant trunk in the true lumen and patency of the reimplanted vessels. The dissected KD was still patent secondary to retrograde left subclavian flow and antegrade true lumen flow around the elephant trunk anastomosis. To fix this, the patient underwent a second endovascular procedure.

A 10-mm balloon was inserted from the femoral access into the ALSA and inflated in the true lumen at the origin of the left vertebral artery to facilitate the passage of a guidewire from the axillary artery into the left vertebral artery. Using this guidewire, 2 overlapping covered stents were positioned from the left subclavian artery into the left vertebral artery, redirecting blood away from the KD. The dissected subclavian artery, supplying the KD's false lumen, was embolized using a spiral Penumbra packing coil and occluded with a 14-mm plug.

Finally, intravascular ultrasound guided the creation of a longitudinal fenestration with cheese wire technique, accommodating and excluding the false channel with 2 stents.

The final angiogram showed complete exclusion of the false lumen, no endoleak, and regular perfusion of both the vertebral artery and visceral branches. The postoperative course was uneventful, with good distal perfusion and no signs of ischemia or neuromuscular deficits.

## Discussion

RSAA is a rare condition, usually asymptomatic, with manifestations in about 5% of cases, typically resulting from compression of surrounding structures.^1^ KD rupture is between 6% and 19%, and the risk of dissection ranges from 20% to 50%.^2^^,^E3, E4, E5, E6 Surgical intervention becomes crucial in cases complicated by persistent hypertension, recurrent pain, rupture, malperfusion, or aortic expansion.^3^ In our case, the large KD and persistent pain necessitated urgent surgery, planned collaboratively between cardiac and vascular surgeons and interventional radiologists.

Open surgical repair is the gold standard for aortic arch pathology at our institution.E7, E8, E9 However, the complexity of this case precluded a one-stage frozen elephant trunk surgery or single endovascular repair, leading us to employ innovative techniques.

A soft prosthesis was chosen to reduce the risk of placing a rigid TEVAR near the area of anatomical contact between the aorta and esophagus, keeping the overlapping area as far away from this region as possible.

Reaching the left subclavian artery via full median sternotomy was complicated by the aorta's course and the left subclavian pathway behind the pulmonary hilum. Some authors recommend thoracotomy for RSAA repairs.^4^^,^^E10^ However, in this patient. given the anatomical complexity, it was not an adequate access in order to fully treat the pathology.

Since the Society for Vascular Surgery guidelines recommend a subclavian bypass to ensure good arm perfusion in patients undergoing thoracic endovascular aortic repair,^5^ we performed an extra-anatomical aortoaxillary bypass, followed by left subclavian embolization, to prevent backflow into the dissected KD. This approach effectively prevents blood flow into the aneurysmal segment, reducing the risk of further dissection or rupture. Preservation of the left vertebral artery was crucial because of the contralateral artery's underdevelopment. We used an endovascular bypass (we called it “banana bypass”) to connect the vertebral artery to the distal subclavian artery, safely excluding the proximal aneurysm.

Our approach also involved aortic fenestration using the “cheese wire” maneuver to alleviate malperfusion, paired with a thoracic stent-graft to exclude the dissected aorta while minimizing risks of compression or erosion the trachea and esophagus.E11, E12, E13

In high-risk patients, hybrid repair with total debranching and thoracic endovascular repair can be considered, though it carries the risk of fistula formation near the esophagus and trachea.[Bibr bib19][Bibr bib20]

## Conclusions

The 2-step hybrid approach combining soft elephant trunk and endovascular completion provides an effective treatment for complex aortic pathologies like RSAA and KD but requires high surgical skills, a multidisciplinary team and an experienced center. The “banana bypass” preserves blood flow to the vertebral artery while safely excluding the proximal aneurysm. The classical “soft” elephant trunk remains a vital treatment option, providing valuable solutions for both surgeons and patients.

## Conflict of Interest Statement

The authors reported no conflicts of interest.

The *Journal* policy requires editors and reviewers to disclose conflicts of interest and to decline handling or reviewing manuscripts for which they may have a conflict of interest. The editors and reviewers of this article have no conflicts of interest.

## Supplementary Data

Video 1 Step by step description of the hybrid surgical procedure. Video available at: https://www.jtcvs.org/article/S2950-6050(25)00003-8/fulltext.
