# Supplementary figures and images for: Hybrid repair with soft elephant trunk for acute type B dissection in a patient with right-sided aortic arch and Kommerell diverticulum
Source: JTCVS Struct Endovasc. 2025 Jan 27;5:100044. doi: 10.1016/j.xjse.2025.100044 (PMC13244680; doi:10.1016/j.xjse.2025.100044)

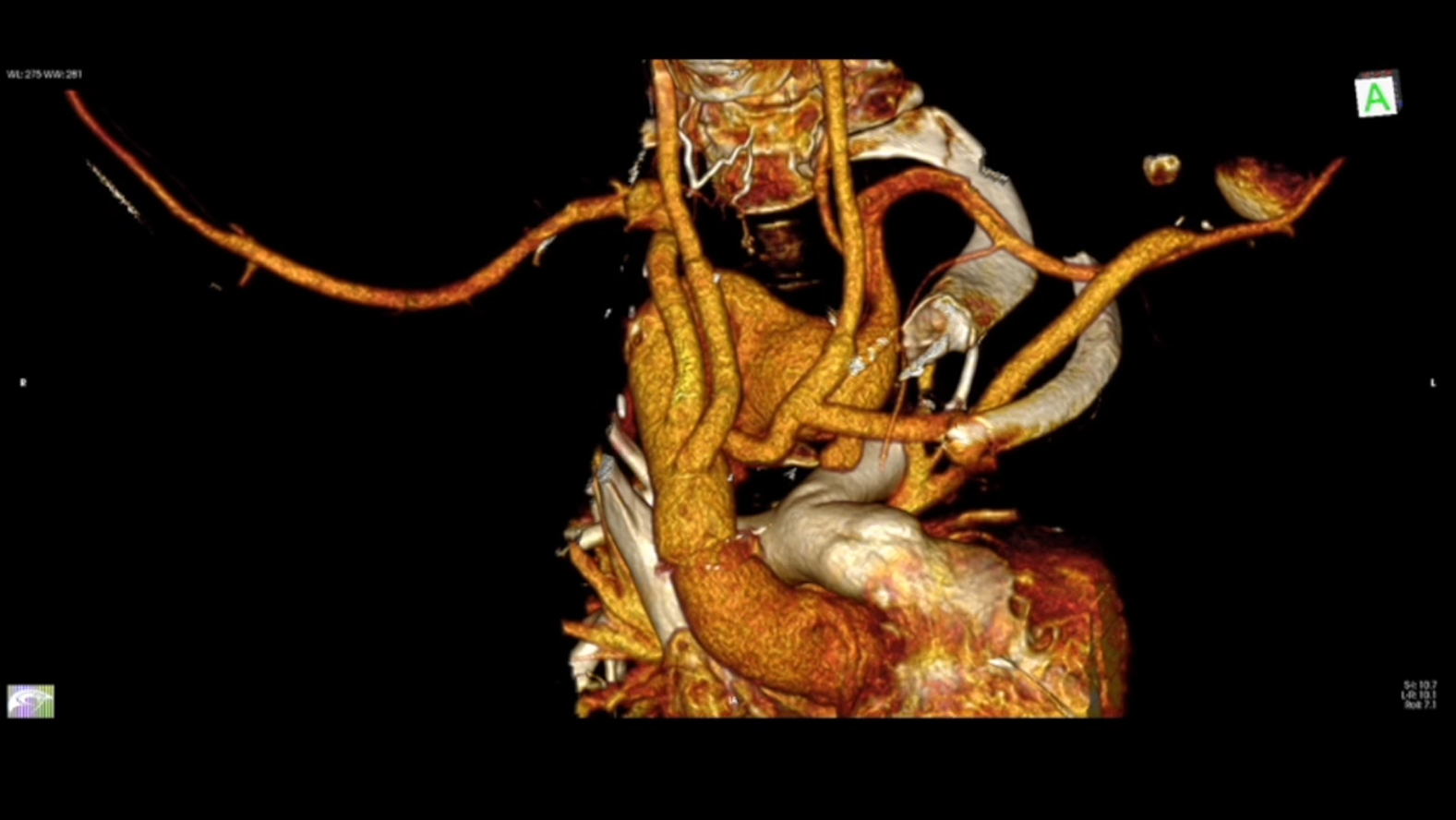

Supplement: Video 1 — Step by step description of the hybrid surgical procedure. Video available at: https://www.jtcvs.org/article/S2950-6050(25)00003-8/fulltext. [file fx2.jpg]
